# Early Exposure to Step 1-Style Questions in the Preclinical Curriculum: A Student-Led Initiative to Support Clinical Reasoning and United States Medical Licensing Examination (USMLE) Preparation

**DOI:** 10.7759/cureus.95020

**Published:** 2025-10-20

**Authors:** Molly Courtright, Hunter D Alexander, Chloe Eckert, Rachel Schwyhart, Abigail Camenisch, Hannah Godfrey, Zachary Leibovit-Reiben, Richard Amini

**Affiliations:** 1 Medicine, University of Arizona College of Medicine - Tucson, Tucson, USA; 2 Dermatology, Mayo Clinic Arizona, Scottsdale, USA; 3 Dermatology, University of Arizona College of Medicine - Tucson, Tucson, USA; 4 Emergency Medicine, University of Arizona, Tucson, USA

**Keywords:** board exam, customized medical education, education and training of medical students and doctors (specialist and phd)), medical education curriculum, medical school education, online medical education, student education, united states medical licensing examination (usmle), usmle step 1 pass-fail

## Abstract

Preclinical medical students often face challenges transitioning to United States Medical Licensing Examination (USMLE) Step 1-style board questions due to differences in content emphasis and question format compared to traditional coursework. Through a student-led initiative, weekly AMBOSS question sets with explanatory videos were created to align with the University of Arizona College of Medicine - Tucson’s (UACOM-T) curriculum, with the goal of familiarizing first-year students with board-style questions early in their training. To assess the perceived usefulness and impact of this resource, a survey was distributed to preclinical students, which gathered feedback on engagement, perceived benefits, and suggestions for improvement after six months of use. Survey responses were collected from 93 first-year medical students (77% of the class of 2028, referring to students who began in 2024 and are expected to graduate in 2028), with 90.3% (95% CI: 82.6%-94.8%) of respondents using at least some of the weekly question sets. About 92.3% (95% CI: 85.3%-96.3%) of respondents felt more prepared for board-style questions because of these sets. And 73.2% (95% CI: 63.3%-81.1%) of respondents also stated that the sets increased their confidence in answering board-style questions. About 82.8% (95% CI: 73.9%-89.1%) also felt it enhanced their understanding of the preclinical curriculum. These initial results indicate that most first-year students engaged with the question sets and gained confidence in applying their preclinical knowledge to Step 1-style questions.

## Introduction

The existing curriculum at medical institutions in the United States, including the University of Arizona College of Medicine - Tucson (UACOM-T), is structured to support excellence in medical knowledge and clinical training. At the same time, this training includes performing well on national licensure exams. It is known that students benefit from complementary resources targeting the United States Medical Licensing Examination (USMLE) Step 1 content while preparing for these exams [1,2]. However, medical students often encounter challenges when transitioning from their institution’s preclinical curriculum to USMLE Step 1 preparation, including increased stress during dedicated study periods even after the 2022 transition of Step 1 to Pass/Fail [3-5].

Literature suggests that regular and early engagement of commercial USMLE Step 1-style question banks (i.e., AMBOSS and USMLE World) is associated with higher USMLE Step 1 scores, independent of other academic predictors such as MCAT (Medical College Admission Test) scores or preclinical grades [6-9]. However, there is limited data regarding student-perceived preparedness for USMLE board exams after using these external resources. One project providing class-wide access to a commercial Step 1 question bank during preclinical modules found that consistent, longitudinal use improved both performance on USMLE Step 1 and perceptions of readiness [8]. However, this data is based on self-made question sets and does not correlate with any institution-based content.

The objective of this feasibility study was to integrate student-selected USMLE Step 1-style questions from the AMBOSS question bank into the preclinical curriculum to assess whether early question exposure increases student confidence regarding USMLE board exams. UACOM-T follows a system-based preclinical curriculum, allowing for multisystem integration of subjects that mirror USMLE Step 1-style questions. The major goals of this project were to (1) increase confidence and perceived preparedness in approaching USMLE board questions, (2) integrate USMLE board content into existing preclinical coursework, and (3) model clinical reasoning and test-taking approaches for board exams.

## Materials and methods

Study design and setting

This was a single-center cross-sectional study conducted at the University of Arizona, College of Medicine - Tucson. This study was reviewed by the University of Arizona Institutional Review Board (Submission ID: STUDY00006705) and approved as a Quality Improvement (QI) study, which does not constitute human subjects research. The study participants were 121 medical students during their first year of medical school. Participation in the study was voluntary.

Because this project was conducted as a QI initiative with primarily qualitative outcomes, an a priori sample size calculation was not performed. Instead, the sample size was determined by the number of eligible participants during the study period. Of the 121 first-year medical students, 93 completed the survey, yielding a response rate of 77%. This high level of participation ensured adequate representation of the target population and allowed for meaningful analysis.

Existing curriculum

UACOM-T utilizes a three-phase curriculum, including an 18-month preclinical phase with eight integrated system-based blocks covering normal and abnormal physiology, a 12-month clerkship phase with eight core clerkships, and a 14-month transition to residency phase with sub-internships/electives. The first year (M1) consists of five system-based blocks within the preclinical phase: foundations, musculoskeletal system, nervous system, cardiovascular/pulmonary/renal systems, and digestion/metabolism/hormones (DMH).

As part of the learning resources provided to all UACOM-T students, they receive a subscription to AMBOSS (Berlin, Germany), an online medical learning platform that integrates an extensive question bank with a searchable medical knowledge library. The platform includes more than 5,000 USMLE-style multiple-choice questions, each linked to detailed reference content, evidence-based explanations, and multimedia resources. AMBOSS questions are rated for difficulty on a scale from 1 to 5.

Intervention

The educational intervention consisted of collaborating with third- and fourth-year medical students and block directors at UACOM-T to curate an optional set of 10 to 20 AMBOSS questions each week, selected to match the specific learning objectives of their block during the preclinical phase of the curriculum.

Curation of questions

Volunteer third- and fourth-year medical students at UACOM-T who passed Step 1 on their first attempt (called AMBOSSadors) were provided with learning objectives, lecture notes, and lecture slides for the 18-month preclinical curriculum several months prior to the beginning of each preclinical block. Blocks were then divided by the group, with certain students covering each block. AMBOSSadors identified questions that tied directly to in-house lecture learning objectives by sequentially reviewing question banks to ensure correlation. Initially, questions with lower difficulty (one to two hammers) were selected to ease students into board-style questions. As the academic year progressed, questions with one to four hammers were included based on the discretion of the student and question availability. Once the question sets were created, the individual faculty block directors reviewed the sets and kept or removed questions based on their perceived alignment with learning objectives. The majority of questions were used from each set submitted, resulting in a set of 10-20 questions covering about 40 hours of content that week. Following approval, the student curated a recorded video guiding viewers through their clinical reasoning for each question in the set posted on the school's learning platform, MedLearn.

Assessment

Assessment consisted of an anonymous electronic survey distributed to all first-year medical students at UACOM-T to assess perceived USMLE question confidence and understanding of preclinical content. Questions were created by the AMBOSSadors to quantify subjective use of the questions by students and identify areas for improvement. The survey utilized the Likert scale for reproducibility. The survey was sent via email and presented via a QR (quick response) code during a required in-person session during the final block of the first year.

Data analysis

The survey data were obtained through Google Forms; no identifying information was collected from the students. Data were exported into Microsoft Excel (Redmond, WA) and imported into R (Auckland, New Zealand), a statistical computing software. Results are presented as means and percentages with 95% confidence intervals. A paired t-test was used for comparison of paired samples. The statistical level of significance was set at p < 0.05.

## Results

Out of the 121 first-year medical students at the UACOM-T, 93 completed the survey after six months of exposure to the weekly AMBOSS question sets (77% response rate). Nearly all the respondents, 90.3% (95% CI: 82.6%-94.8%) engaged with the question sets in some capacity; 43.0% (95% CI: 33.4%-53.2%) reported completing all or most of the sets, and 65.6% (95% CI: 55.5%-74.5%) used the sets prior to curricular block exams, with 17.2% (95% CI: 10.9%-26.1%) completing them weekly.

Students responded that the question sets are a valuable tool for enhancing their understanding of preclinical content and aligning learning with Step 1-style application. About 82.8% (95% CI: 73.9%-89.1%) of students agreed or strongly agreed that the sets enhanced their understanding of the curriculum, and 83.9% (95% CI: 75.1%-90.0%) reported that they aligned well with block content.

About 92.3% (95% CI: 85.3%-96.3%) reported feeling more prepared for Step 1-style questions after using the weekly question sets. Confidence in approaching Step 1-style questions increased for 73.2% (95% CI: 63.3%-81.1%) of students.

Regarding associated stress and the question sets, 40.9% (95% CI: 31.4%-51.0%) reported neutral changes, 29.0% (95% CI: 20.8%-38.9%) reported somewhat increased changes, and 18.3% (95% CI: 11.7%-27.3%) reported greatly increased stress.

Table 1 presents the patterns of student engagement with the question sets, detailing both the frequency of completion and the manner in which they were utilized.

**Table 1:** Engagement patterns

Response Category	% of Respondents
Q1: How many of the weekly question sets have you completed?	
All weekly sets so far	22.6% (95% CI: 15.3%-32.1%)
Most weekly sets (75% or more)	20.4% (95% CI: 13.5%-29.7%)
Some weekly sets (25%–75%)	23.7% (95% CI: 16.2%-33.2%)
Few weekly sets (<25%)	23.7% (95% CI: 16.2%-33.2%)
None	9.7% (95% CI: 5.2%-17.4%)
Q2: How did you typically use the question sets? (Select all that apply)	
Completed sets prior to block exams	65.6% (95% CI: 55.5%-74.5%)
Completed sets weekly	17.2% (95% CI: 10.9%-26.1%)
Completed sets + videos prior to exams	4.3% (95% CI: 1.7%-10.5%)
Completed sets + videos weekly	3.2% (95% CI: 1.1%-9.1%)
Other (miscellaneous responses)	9.7% (95% CI: 5.2%-17.4%)
Q3: Did you complete the question sets independently or in a group setting?	
Independently	62.4% (95% CI: 52.2%-71.5%)
Both independently and in group	31.2% (95% CI: 22.7%-41.2%)
Group setting only	6.5% (95% CI: 3.0%-13.4%)

Table 2 presents students’ perceptions of the effectiveness of the question sets, including their impact on understanding of the curriculum, the degree of alignment with the college’s content, and the value of the accompanying explanatory videos.

**Table 2:** Perceived effectiveness of weekly question sets

Response Category	% of Respondents
Q1: The weekly question sets enhanced my understanding of the curriculum.	
Strongly agree	36.6% (95% CI: 27.5%-46.7%)
Agree	46.2% (95% CI: 36.5%-56.3%)
Neutral	15.1% (95% CI: 9.2%-23.7%)
Disagree	2.2% (95% CI: 0.6%-7.5%)
Q2: The weekly question sets aligned well with the weekly content covered in the block.	
Strongly agree	32.3% (95% CI: 23.6%-42.3%)
Agree	51.6% (95% CI: 41.6%-61.5%)
Neutral	14.0% (95% CI: 8.4%-22.5%)
Disagree	2.2% (95% CI: 0.6%-7.5%)
Q3: The explanatory videos improved my ability to approach Step 1-style questions.	
Strongly agree	8.6% (95% CI: 4.4%-16.1%)
Agree	19.4% (95% CI: 12.6%-28.5%)
Neutral	9.7% (95% CI: 5.2%-17.4%)
Did not use videos	62.4% (95% CI: 52.2%-71.5%)

Table 3 presents the impact of the question sets on perceived Step 1 preparedness, confidence, and exam-related stress levels.

**Table 3:** Step 1 preparation and stress outcomes

Response Category	% of Respondents
Q1: Do you feel more prepared for Step 1-style questions because of these question sets?	
Yes	92.3% (95% CI: 85.3%-96.3%)
No	7.7% (95% CI: 3.7%-14.7%)
Q2: Since using the weekly question sets, how has your confidence in answering Step 1-style questions changed?	
Greatly increased	19.4% (95% CI: 12.6%-28.5%)
Somewhat increased	53.8% (95% CI: 43.7%-63.5%)
Neutral	23.7% (95% CI: 16.2%-33.2%)
Somewhat decreased	3.2% (95% CI: 1.1%-9.1%)
Q3: Since using the weekly question sets, how has your stress about preparing for Step 1 changed?	
Greatly decreased	1.1% (95% CI: 0.2%-5.8%)
Somewhat decreased	10.8% (95% CI: 5.9%-18.7%)
Neutral	40.9% (95% CI: 31.4%-51.0%)
Somewhat increased	29.0% (95% CI: 20.8%-38.9%)
Greatly increased	18.3% (95% CI: 11.7%-27.3%)

Table 4 outlines the qualitative feedback on the project, highlighting both areas for improvement and the benefits students reported from the question sets. Repeated comments regarding areas for improvement are listed once, with the number of responses indicated in parentheses.

**Table 4:** Qualitative feedback

Responses
I love these practice sets and feel quite grateful that the time was taken to narrow down questions that apply to our current learning. I often feel like there is a lack of application questions in our curriculum that prepare us for Step 1. Love these!
I liked that the questions were curated for the relevant content that we were learning that week.
Applying what I learned that week to questions and reinforcing concepts helps me to master the content.
I enjoyed the act of testing knowledge and written explanations.
I think it’s helpful to review the concepts in different clinical vignettes than the ones we get in class.
I liked how relevant the content was for the block. It aligned well.
Prepares me to think more critically and focus on less memorization and info regurgitation and more understanding the big picture.
I like that it helps me gauge the areas I still need to focus on and what areas I am understanding well.
I like completing the questions a few days before the exam. Extra practice questions.
I love using questions to learn as I’ve found that it’s the most effective form of studying for me, so these sets have been so nice! Thank you so much!
I think the fact that they applied to our class information helped to reinforce a lot.
Sometimes I feel that certain blocks don’t include enough practice questions for the exams, and I feel like these question sets have been very helpful.
I think these are amazing practice tools. Thank you so much.
THIS IS SO HELPFUL, THANK YOU! Specifically it's most helpful that the questions align with class material.
I liked that they are relevant to the content we are learning!
I think they’re a great way to gauge if I pulled important points from the lectures.
I’m grateful they exist! The videos are helpful.
It is a great way of synthesizing all the clinical concepts into a clinical vignette. And it was very helpful and encouraging regarding my ability to do well on these types of questions/STEP. I do much better on AMBOSS questions than I do on block exams/questions.
I think it was helpful mainly to start practicing with Step 1-style questions, particularly in how the information is presented.
It was much more engaging by way to test my knowledge and review concepts. I struggle to use anki, so this is so helpful to review content without consuming my life.
They were extremely helpful in reviewing major concepts before block exams!
The attached video explanations were very helpful.
I like that they center my understanding and add clinical context.
Including more questions in the sets (13 responses).
Wanting more regular reminders about the sets (2 responses).
Not finding the videos helpful (4 responses).

## Discussion

In summary, this six-month project provides evidence that embedding USMLE Step 1-style questions longitudinally within the preclinical curriculum can positively impact student preparedness and confidence regarding USMLE board exams. A student-led model with faculty collaboration offers a scalable and adaptable approach to supporting board preparedness, and ongoing iterative refinement based on student feedback will be critical to maximizing its impact.

It has been well established that early, distributed practice with board-style questions improves performance on USMLE exams [6-8]. It is suggested that integrating board-style questions into the preclinical curriculum promotes more efficient learning and self-regulated study habits, hopefully leading to success on USMLE exams [10-13]. A strong correlation has also been found between student-perceived readiness and exam performance on in-house exams [14]. However, few studies have looked at the factors affecting perceptions of readiness. Our study aimed to investigate the relationship between curriculum-correlated question banks and students’ perceived readiness, both for in-house content and onboard exams.

This six-month pilot demonstrated that longitudinal exposure to Step 1-style questions within the preclinical curriculum increased perceived preparedness (92.3% (95% CI: 85.3%-96.3%)) and confidence (73.2% (95% CI: 63.3%-81.1%)), aligning with our primary goal. A key benefit of incorporating weekly questions throughout the curriculum is that it combines external board exam resources with our college’s traditional in-house materials, creating a balance between dedicated board preparation and content aimed at building strong clinical competence.

We found that 90.3% (95% CI: 82.6%-94.8%) of students engaged with the question sets, while only 9.7% (95% CI: 5.2%-17.4%) reported completing none. Given that a large proportion of students are already utilizing the question sets voluntarily, formal integration into the curriculum could be pursued in the future as the students have already proven their interest in completing them and would standardize preparation and further reinforce learning across the cohort.

The free-response survey answers were particularly helpful, as they highlighted consistent student enthusiasm for the weekly question sets and added valuable context to the quantitative results. Students frequently emphasized that the sets complemented their block content, reinforced key concepts, and provided opportunities to apply knowledge in a different format. Many noted that the clinical vignette style encouraged critical thinking, synthesis of information, and a deeper understanding beyond memorization. The sets were also valued as a tool for self-assessment, allowing students to identify strengths and areas for improvement, and several described them as one of the most effective and engaging study strategies compared to other methods. Suggestions for improvement included adding more questions per set and providing more consistent reminders. Taken together, the qualitative feedback underscored that the question sets complemented existing coursework, supported exam preparation, and encouraged a more meaningful, application-focused approach to learning.

Representative comments included:

“I love these practice sets and feel quite grateful that the time was taken to narrow down questions that apply to our current learning. I often feel like there is a lack of application questions in our curriculum that prepare us for Step 1. Love these!”

“It’s great to have practice problems readily available to work through. I feel like it completes the picture of disease presentations a lot of the time for me.”

“Applying what I learned that week to questions and reinforcing concepts - they help me to master the content.”

However, this early exposure also appears to have increased subjective stress levels in 47.3% (95% CI: 37.5%-57.4%) of students. The mixed stress responses underscore an important tension inherent to board preparation: Increased exposure to high-stakes exam content may simultaneously build competence and provoke stress. This may reflect students recognizing the extent of material still to be mastered as they completed more questions, or it may stem from inconsistent use of the sets, leading to stress from avoidance or self-comparison with peers. In previous studies, consistent self-assessment seminars were shown to lower average levels of anxiety for students [4]. What is still unknown is whether this stress is short-lived, and if the continued use of this resource would actually decrease stress levels over time with greater exposure.

Our second objective aimed to align USMLE questions with our in-class material. In our study, creating question banks that correlate with the in-house curriculum reduced the need for students to create their own self-assessments, thereby making this resource more accessible compared to other individually made self-assessments used in previous studies [14]. Our data suggests that students found this resource helpful: 83.9% (95% CI: 75.1%-90.0%) reported improved alignment with weekly block content, and 82.8% (95% CI: 73.9%-89.1%) of students reported enhanced understanding of the in-house curriculum. This allows students to use a singular study resource in order to increase their confidence for both board exams and institution-based learning. In addition to alignment with preclinical content, another benefit from this project is that it did not require additional demand from UACOM-T faculty to curate these questions, but rather relied on students who wanted more experience with medical education and teaching.

The third objective was to create videos that modeled an approach to answering Step 1-style questions, including strategies for analyzing clinical vignettes, eliminating distractors, recognizing key patterns, and applying relevant content knowledge. The format aimed to promote not only test-taking skills but also clinical acumen. Our data showed that 62.4% (95% CI: 52.2%-71.5%) of students did not use the videos, though 28.0% (95% CI: 19.9%-37.8%) of users found them helpful. Due to the limited engagement, we cannot comment on the efficacy of this aspect of the project. However, because the videos require considerable time and effort from the student ambassadors to produce, we are re-evaluating their role and considering how best to integrate or adapt them in future iterations.

This project leverages the existing AMBOSS subscription purchased by UACOM-T, ensuring that all students have equitable access to high-quality study materials, an approach that may be especially valuable for schools in rural regions or those serving students from diverse socioeconomic backgrounds. Prior research has shown that students from lower socioeconomic backgrounds often enter medical school with lower average MCAT scores but are able to achieve comparable performance through Step 2 [15], a trend attributed in part to standardized curricula and equal access to resources. Therefore, the universal availability of AMBOSS in our study may help mitigate disparities in study resource access.

Looking forward, students frequently suggested increasing the number of questions in each set. While we initially limited sets to 10-20 questions per week to avoid overwhelming already demanding schedules, we plan to expand future sets to 40-60 questions weekly. Additional feedback included sending more frequent reminders and providing clearer instructions for accessing the sets, changes we also intend to implement. Given how many students expressed that they found the sets helpful and the enthusiasm surrounding their use, we are also considering making them a required component of the curriculum to ensure all students can benefit.

The most exciting avenue for future research involves looking into the effects of this initiative on how longitudinal exposure to these questions impacts preclinical grades, length of the Step 1 dedicated study period, Step 1 pass rates, frequency of Step 1 delays, clinical shelf exam scores, and Step 2 performance. This will help determine whether early, curriculum-aligned exposure to external USMLE board preparation resources influences board outcomes without major faculty investment. If future studies demonstrate improved performance, this model could be adopted by other medical schools to support their students at very low faculty cost and time requirements. Further innovation and research should be pursued to evaluate the efficacy of these types of interventions.

Limitations 

This study has several limitations, including that the results were based on subjective student responses. Furthermore, not all students responded to the questionnaire. Student-reported feedback also highlighted the need for sets with more questions, as not all relevant USMLE topics were included due to restrictions on the number of questions per set. Additionally, data was not collected regarding the video explanations; therefore, we do not know why this aspect was not utilized to the same degree. The survey questions were not validated, leading to gaps in data acquisition. Although this may impact reproducibility, survey questions were included in the study.

Another limitation is that the data included a small sample size from only one medical institution. Additionally, this project relied on volunteer student leaders to curate question sets with different students covering individual blocks. This increased variability between types of selected questions between blocks. Another element of this variability was the AMBOSS hammer difficulty selected based on student discretion, leading to irregularities in the difficulty of the assessment. Because the AMBOSSadors were volunteer students, there was no rigorous selection process. Lastly, our study was not designed to assess overall performance on the USMLE Step 1 exam, limiting our ability to directly attribute academic success onboard exams to this intervention; however, we hope to continue collecting data in order to assess this in the future.

## Conclusions

This six-month pilot integrating weekly Step 1-style questions, correlated with in-house preclinical material, for first-year medical students suggests that early, consistent exposure can improve students’ perceived preparedness and confidence for board exams. This student-led, faculty-supported format ensured strong alignment with institutional content while providing a sustainable model that did not add significant faculty workload.

Survey feedback highlighted strong engagement and appreciation for the resource, along with clear opportunities for improvement, including increasing the number of questions and enhancing accessibility through regular reminders. While some students reported heightened stress, others valued the early exposure as a way to identify knowledge gaps. Future work should explore whether these perceptions translate into improved academic and board exam performance.
